# Modeling methylation data as an additional genetic variance component

**DOI:** 10.1186/s12919-018-0128-7

**Published:** 2018-09-17

**Authors:** Marcio Almeida, Juan Peralta, Jose Garcia, Vincent Diego, Harald Goring, Sarah Williams-Blangero, John Blangero

**Affiliations:** 1 South Texas Diabetes and Obesity Institute, University of Texas Rio Grande Valley School of Medicine, One West University Blvd., STDOI Modular Building #100, Brownsville, TX 78520 USA; 2 Menzies Institute for Medical Research, University of Tasmania, 17 Liverpool Street, Hobart, TAS Australia

## Abstract

High-throughput platforms allow the characterization of thousands of previously known methylation sites. These platforms have great potential for investigating the epigenetic effects that are partially responsible for gene expression control. Methylation sites provide a bridge for the investigation of real-time environmental contributions on genomic events by the alteration of methylation status of those sites. Using the data provided by GAW20’s organization committee, we calculated the heritability estimates of each cytosine-phosphate-guanine (CpG) island before and after the use of fenofibrate, a lipid-control drug. Surprisingly, we detected substantially high heritability estimates before drug usage. This somewhat unexpected high sample correlation was corrected by the use of principal components and the distributions of heritability estimates before and after fenofibrate treatment, which made the distributions comparable. The methylation sites located near a gene were collected and a genetic relationship matrix estimated to represent the overall correlation between samples. We implemented a random-effect association test to screen genes whose methylation patterns partially explain the observable high-density lipoprotein (HDL) heritability. Our leading association was observed for the *TMEM52* gene that encodes a transmembrane protein, and is largely expressed in the liver, had not been previously associated with HDL until this manuscript. Using a variance component decomposition framework with the linear mixed model allows the integration of data from different sources, such as methylation, gene expression, metabolomics, and proteomics. The decomposition of the genetic variance component decomposition provides a flexible analytical approach for the challenges of this new omics era.

## Background

The identification of reliable genetic factors associated with phenotypes of interest is the major goal of large genetic and epidemiologic projects. The immense number of multiple hypotheses tested and the confounding effects of environmental contribution jeopardize this identification [1]. The correct modeling of the environmental contribution to a trait of interest is very challenging as it is almost impossible to control modeling of the environmental contribution in study design using human samples. The assessment of genome-wide methylation patterns provides an interesting bridge to understanding the epigenetic effects and, consequently, the environmental contribution to any phenotypes being studied. Highly methylated DNA sequences have a significant repressive role in the control of the expression of nearby genes. The presence of methylated terminals on those sites represses the transcription machinery assembly and, consequently, the expression of a target gene. The fine control of gene expression has a considerable impact on messenger RNA transcription and, consequently, on protein production. Genome-wide methylation platforms enable the simultaneous analysis of hundreds of thousands of suitable methylated sites that are dispersed across the human genome. This type of data has been used by various epigenetic-wide association studies for the identification of differentially methylated cytosine-phosphate-guanine (CpG) sites associated with diseases, clinical outcomes, environmental exposures, or other experimental conditions [2]. Despite their clear potential, the incorporation of methylation data in a genetic analytical framework is still debatable and open for alternative approaches [3].

The GAW20 challenged the scientific community to propose and test different analytical methods to be applied at the genetic and epigenetic data shared by the Genetics of Lipid Lowering Drugs and Diet Network (GOLDN) initiative [4, 5]. The status of methylation sites near genes were collected and for each gene we calculate a pairwise genetic relationship matrix (GRM) between samples. These gene-specific GRMs represent the covariance between methylation sites surrounding a gene and are a simplified representation of the transcriptional control acting on this gene. These gene-specific GRMs were tested as additional genetic variance components to explain phenotypic variability for the real high-density lipoprotein 2 (HDL2) phenotype as shared by GAW20 organization [5]. Gene-specific GRMs responsible for a considerable proportion of HDL phenotypic heritability were selected. No GRM-based association tested reached the desired multiple hypothesis corrected threshold and the best candidate identified was the gene *TMEM5* using the methylation data before use of fenofibrate. This candidate gene encodes a transmembrane protein and was implicated with the liver enzymatic repertoire, but until our study, it had not been associated with hypercholesteremia. Genetic variance component decomposition offers the required flexibility to incorporate different sources of biological data [6]. The biological data integration is challenging, but, at the same time, has great potential to improve our understanding of the different molecular layers acting in a phenotype of interest.

## Methods

### Heritability estimation of methylated genomic sites

The additive heritability estimate was calculated for each methylated site using the polygenic routine implemented in SOLAR [7]. The methylation sites were inverse-normalized and before and after fenofibrate heritabilities were estimated on each site and their distributions were compared to evaluate overall differences in genome-wide methylation patterns resulting from the fenofibrate use.

### Principal component analysis of methylation data

The complete set of inversed-normalized methylation sites was randomized and a subset of 10% of them was selected. Principal components were estimated, using native R implementation, for the selected subset of methylation sites [8]. The first 20 principal components were obtained and used to decorrelate the methylation data before and after the use of fenofibrate.

### Calculation of gene-specific GRM using methylation data

Using the annotation data provided by GAW20 organization, we defined the complete set of methylation sites mapped to each gene. This information was used as input for an *in-house* program that calculates the correlation between individuals based on the status of their respective methylation sites for each gene. The gene-specific methylation sites collected were standardized and a matrix *Z* was defined. A covariance matrix *R* was derived from *Z* as *R* = *Z* * *Z*^*T*^ where *Z*^*T*^ is the transpose of *Z*. A scaling transformation was applied to *Z* to ensure that all diagonal elements equaled 1. The resulting matrix, *K*, was our gene-specific covariance kernel [9]. The kernels represent the pairwise sample covariance estimates and these matrices are introduced as additional variance components of phenotypic variability into a linear mixed model [6].

### Linear mixed model with an additional methylation component

A new variance component parameter was added into a standard pedigree-based variance component model, $\varOmega \kern0.5em =\kern0.5em {\sigma}_{Total}^2\left(2\phi {h}_r^2\kern0.5em +2{Eh}_{meth}^2+{Ie}^2\right)$, where Ω is the phenotypic covariance matrix; ${\sigma}_{Total}^2$ is the total phenotypic variance; nd ${h}_r^2$, ${h}_{meth}^2$, and *e*^2^, respectively, represent the proportion that can be attributed to the residual additive effect of polygenes, the gene-specific methylation kernel effect, and a random environmental effect. Several critical structuring kernels are employed to model the covariances between individuals: *Φ* is the expected kinship matrix integrated from the pedigree; *E* is the empirically estimated gene specific methylation GRM; and *I* is the identity matrix. Such kernel-based approaches to test the combined effect of multiple genetic variants were proposed decades ago [10] and have grown in popularity recently [11, 12].

Maximum likelihood estimates (assuming a multivariate normal probability density) and likelihood ratio test (LRT) of the ${h}_{meth}^2$ parameter was obtained using an extension of the *polygenic* command in SOLAR, independently for each gene-specific GRM. The significance of each GRM was obtained by LRT using a null model $\Omega \kern0.5em =\kern0.5em {\sigma}_{Total}^2\left(2\upphi {h}_r^2+{Ie}^2\right)$ as reference. Because the variance component ${h}_{meth}^2$ is tested on its boundary, the LRT statistic is distributed as a 50:50 mixture of a 1-degree-of-freedom chi-square and a point-of-mass zero, although this is conservative. For both models, we calculated the heritability of HDL measured on the second and fourth collection, which matches the methylation data collection. We used sex and age covariates on the linear mixed model.

## Results

### Statistical properties of the genome-wide methylated sites

**Fig. 1:**
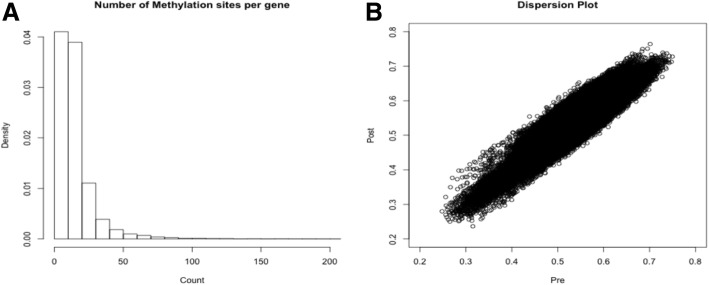
**a** Histogram of the number of methylation sites linked genes; **b** scatterplot of mean intensities of methylation sites before and after fenofibrate treatment

**Fig. 2:**
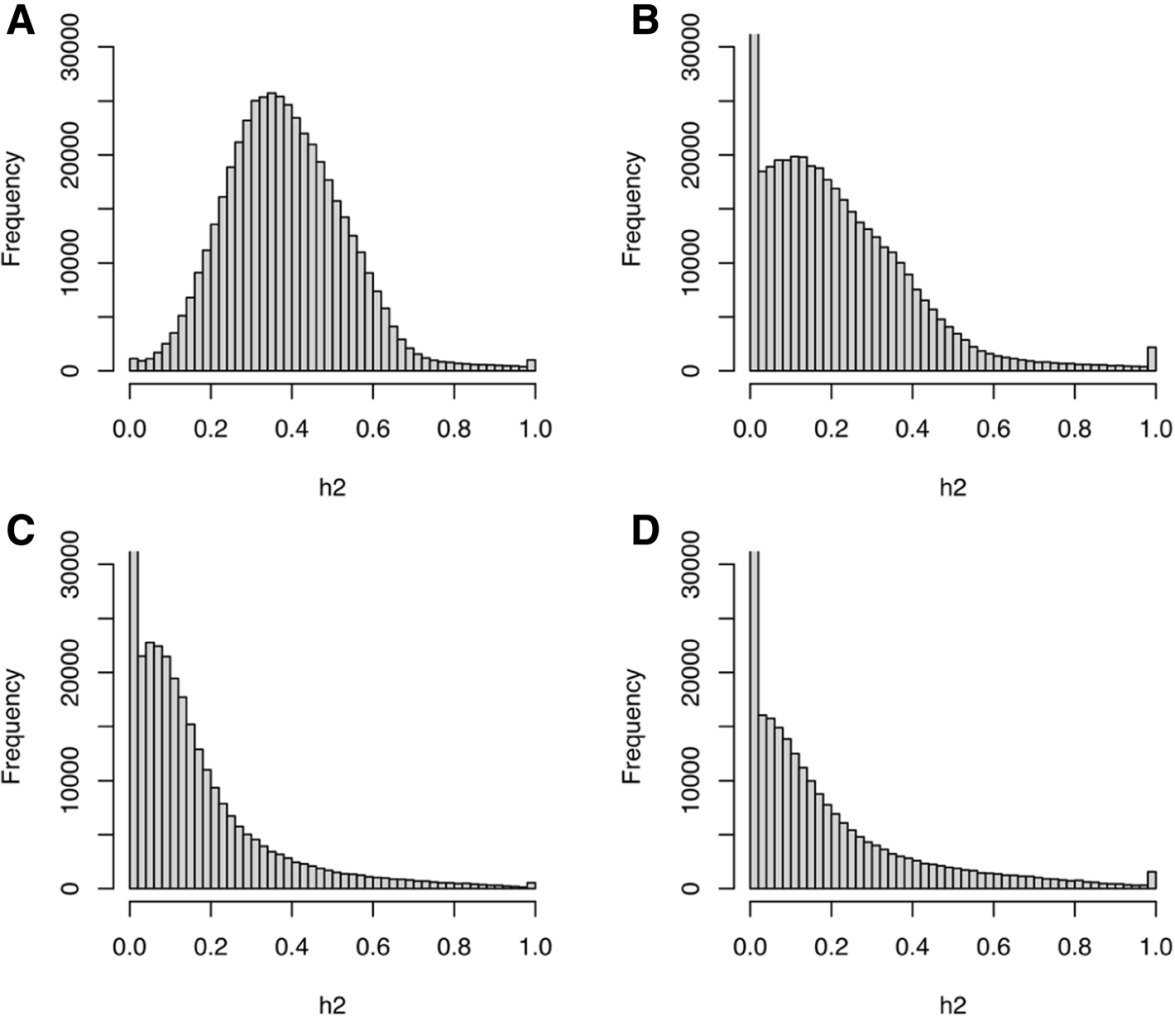
Histogram methylation site heritability estimates. **a** Before fenofibrate treatment. **b** After fenofibrate treatment. **c** Before fenofibrate drug treatment corrected by PC analyses. **d** After fenofibrate treatment correct by PC analyses

A set of 463,996 unique methylation sites were shared by the GAW20 organization, and using the annotation provided, we defined a subset of 349,755 sites linked to at least 1 gene. The methylation data was collected in a set of 990 individuals before fenofibrate usage and 520 individuals after the lipid drug treatment. We identified 22,313 genes with at least 1 methylation site and 80% of the genes had fewer than 20 methylation sites (Fig. 1a). The mean methylation intensities of each site were compared before and after fenofibrate treatment (Fig. 1b). The correlation between methylation sites pre- and posttreatment was pronounced (Pearson correlation = 0.93). The majority of methylated sites is expected to show low to modest heritability estimates. These sites represent responsive genomic elements and provide the molecular bridge between the environmental contribution and fine gene-expression control. To test this premise, we calculated the narrow sense heritability estimate of each methylation site on the 2 time points shared. The distributions of heritabilities estimates (Fig. 2) showed a substantial difference and, specially, unexpectedly high heritability estimates before fenofibrate treatment (Fig. 2a). We hypothesized that the high heritability estimates were caused by a batch effect, during experimental or data processing. We calculated the first 20 principal components (PCs) of methylation data before and after use of fenofibrate. The PCs were added, as covariates, in the linear model and new heritability estimates were calculated (Fig. 2c and d). The addition of PCs was successful and new *h*^*2*^ distributions were comparable (Fig. 2a and c). The corrected heritability estimates are much closer than the ones observed in similar studies using methylation data [13].

**Fig. 3:**
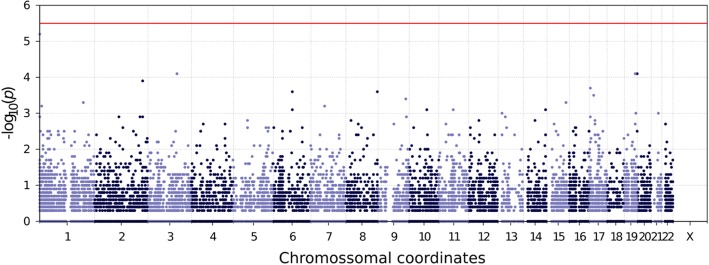
Manhattan plot of gene-specific GRM association results for HDL trait before fenofibrate treatment

**Fig. 4:**
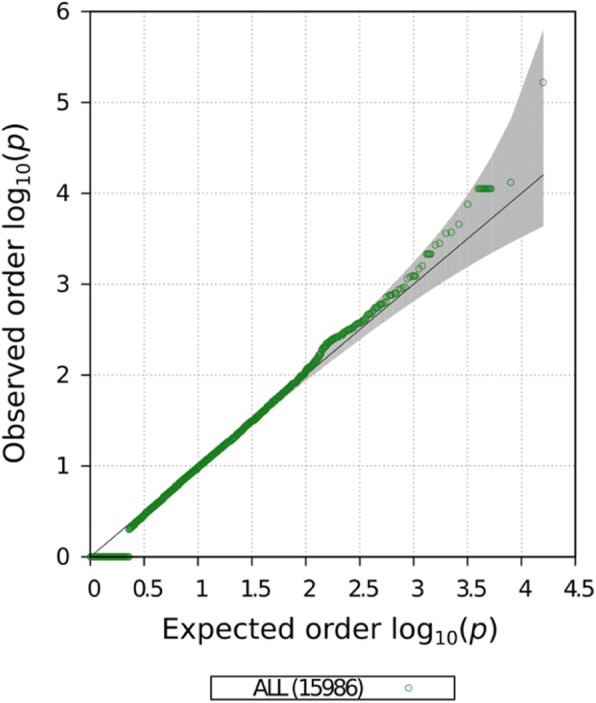
Quantile–quantile (Q-Q) plot of gene-specific GRM association results for HDL trait before fenofibrate treatment

We constructed gene-specific GRM using the PC analysis corrected methylation sites linked to each gene before and after fenofibrate treatment. The covariance GRMs were estimated for a set of 15,596 genes that presented at least 5 methylation sites annotated to it. This minimum number of methylation sites is a requirement to construct valid GRMs as genes with few methylation site measurements could generate high pairwise correlation scores as a result of the lack of information. The contribution of each gene-specific GRM to the phenotypic variability of HDL phenotype was tested using a LRT in the second and fourth time points matching the methylation data collections. No gene-specific–based association tested reached the required multiple hypothesis corrected significance threshold (1.35 × 10^− 6^) in either time point analyzed. The association results for the GRMs calculated before fenofibrate use are presented in Figs. 3 and 4. We identified an interesting association on the gene *TMEM5* (*p* < 5.9 × 10^− 5^). This gene encodes a highly conserved transmembrane protein almost exclusively expressed in the pancreatic tissue, but until our study, had not been associated with HDL blood concentration [14]. The status of methylation sites near *TMEM5* could explain almost 4% of the observed phenotypic variability of HDL. The association statistic distribution was not inflated nor deflated (Fig. 4). The *TMEM5* is an interesting candidate gene but requires an independent replication on similar studies.

## Discussion and conclusions

The correct modeling of genetic and environmental components is a pivotal step in the identification of reliable associations between genetic markers and phenotypes of interest. In general, the environmental component is overlooked and is assumed shared between individuals to simplify the analytical framework applied [4]. Genome-wide methylation arrays were designed to capture the methylation status of thousands of previously detected methylation sites. These platforms measure an active layer of transcriptional control where methylated CpG islands in gene promoters interact with transcription complex machinery and allow a fine control of gene expression. Highly methylated promoter sequences tend to repress gene expression by negatively interacting with nuclear transcription machinery [15]. The gene expression control, mediated by methylation interaction, provide a promising bridge between environment contribution and real-time molecular responses to those insults. Large genetic projects collected epigenetic data to try to identify differentially methylated sites associated with disease states, clinical outcomes, environmental exposures, or other experimental conditions [2, 15]. Despite their potential, the correct modeling for the incorporation of methylation data in a genetic analytical framework is debatable and it became one of the main topics for GAW20 workshop [5].

In this article, we model methylation data using gene-specific GRMs carrying the pairwise samples covariance between methylation sites flanking 15,596 genes dispersed throughout the human genome. The contribution of each GRM for the HDL phenotypic variability was defined using a LRT association test comparing 2 models with and without this added methylation term. We didn’t detect any association that reached the multiple hypothesis corrected significance threshold on either time point. Our best candidate was an interesting association between the gene *TMEM5* and HDL before the use of fenofibrate. Methylation sites flanking this candidate gene were able to explain almost 4% of the trait’s phenotypic variability. These results highlight the relevance of the epigenetic layer of information flanking this gene and its impact on the overall HDL plasmatic concentration. The gene *TMEM5* encodes a Type II transmembrane protein with glycosyltransferase function and is expressed almost exclusively at pancreatic tissue [14, 16]. The *TMEM5* gene has been associated with cobblestone lissencephaly, but it has never been associated with human hypocholesteremia previously [17]. This is a preliminary finding and requires an independent validation [12].

Phenotypic variance component decomposition, the model presented in this article, offers the desired flexibility to combine different sources of information contributing to a phenotype under study [6]. The use of covariance kernels to combine the individual contributions of single-nucleotide polymorphisms has gained a lot of attention lately with the advent of the new cost-effective whole-genome sequencing platforms [11]. These platforms allow the collection of a very dense panel of genetic variations and these alternative approaches are necessary to reduce the burden imposed by the astronomical number of independent statistical tests performed. The combination of different sources of biological data, such as epigenetic, transcriptomic, proteomic, and metabolomics, will improve our understanding of the biological phenomenon. Different sources of biological data can be interpreted as variance components of the observable phenotypic variability of a trait of interest. Combination of this difference sources will improve our knowledge about the molecular players acting on a phenotype and will aid the development of a new generation of personalized drugs.
